# Beverage and Food Fragrance Biotechnology, Novel Applications, Sensory and Sensor Techniques: An Overview

**DOI:** 10.3390/foods8120643

**Published:** 2019-12-05

**Authors:** Alice Vilela, Eunice Bacelar, Teresa Pinto, Rosário Anjos, Elisete Correia, Berta Gonçalves, Fernanda Cosme

**Affiliations:** 1 CQ-VR, Chemistry Research Centre, Department of Biology and Environment, School of Life Sciences and Environment, University of Trás-os-Montes and Alto Douro, P-5000-801 Vila Real, Portugal; fcosme@utad.pt; 2 CITAB, Centre for the Research and Technology of Agro-Environmental and Biological Sciences, Department of Biology and Environment, School of Life Sciences and Environment, University of Trás-os-Montes and Alto Douro, P-5000-801 Vila Real, Portugal; areale@utad.pt (E.B.); tpinto@utad.pt (T.P.); ranjos@utad.pt (R.A.); bertag@utad.pt (B.G.); 3 CQ-VR, Chemistry Research Centre, Department of Mathematics, School of Life Sciences and Environment, University of Trás-os-Montes and Alto Douro, P-5000-801 Vila Real, Portugal; ecorreia@utad.pt; 4 Center for Computational and Stochastic Mathematics (CEMAT), Department of Mathematics, IST-UL, Av. Rovisco Pais 1, 1049-001 Lisboa, Portugal

**Keywords:** Natural flavours, *de novo* synthesis, ligand-receptor interaction, e-nose, *e-tongue*, sensory analysis

## Abstract

Flavours and fragrances are especially important for the beverage and food industries. Biosynthesis or extraction are the two main ways to obtain these important compounds that have many different chemical structures. Consequently, the search for new compounds is challenging for academic and industrial investigation. This overview aims to present the current state of art of beverage fragrance biotechnology, including recent advances in sensory and sensor methodologies and statistical techniques for data analysis. An overview of all the recent findings in beverage and food fragrance biotechnology, including those obtained from natural sources by extraction processes (natural plants as an important source of flavours) or using enzymatic precursor (hydrolytic enzymes), and those obtained by *de novo* synthesis (microorganisms’ respiration/fermentation of simple substrates such as glucose and sucrose), are reviewed. Recent advances have been made in what concerns “beverage fragrances construction” as also in their application products. Moreover, novel sensory and sensor methodologies, primarily used for fragrances quality evaluation, have been developed, as have statistical techniques for sensory and sensors data treatments, allowing a rapid and objective analysis.

## 1. Introduction

The increased consumer preference for natural and sustainable products makes the production of natural flavours, which define the sensory perception of beverages and other food products, an ever-challenging purpose for academic and industrial investigation [1]. Indeed, olfactory signalling initiates when odorant molecules contact the olfactory sensory neurons which express proteins that convert chemical signals into neuronal impulses. Consequently, neurochemical effects occur, in several regions of the brain, generating the smell sense. The discovery that each olfactory neuron expressed one special kind of odorant receptor allowed the researchers Linda Buck and Richard Axel to be distinguished with the Nobel Prize in Medicine in 2004.

Odorant or olfactory receptors (ORs) are located in the human nose, specifically in the olfactory epithelium. However, several studies in recent years had shown the existence of the “ectopic olfactory receptors” in mammals i.e., receptors of the same type spread in many diverse organs and systems [2] such as in tissues as the testes [3] and heart [4]. ORs and related signalling molecules can also be present in non-olfactory areas of the nervous system and may trigger vulnerability to neurodegenerative diseases, and drugs [2].

The olfactory receptors can recognise a myriad of different odour molecules of a diverse protein sequence. According to Farbiszewski and Kranc [5], the olfactory receptors include 172 subfamilies encoded by a single chromosomal *locus*. OR genes constitute the most abundant family of G protein-coupled receptors (GPCRs) with about 1000 genes in the mouse [6]. The human receptor gene family comprises 339 receptor genes and 297 receptor pseudogenes, unequally dispersed in 51 distinct loci on 21 human chromosomes [5]. However, almost half of the human ORs repertoire is non-functional [7], leading to the conclusion that, throughout the process of development, the sense of smell may have lost importance for primates. It is known that the quantity of functional OR genes is lower, in humans, when compared to other species. Nevertheless, humans have an extremely sensitive sense of smell, essential for the discovery of odours that are necessary for maintaining a healthy life, such as the smell of smoke (detection of fire) and the smell of rotten food (to avoid its ingestion) [8].

It is also important to note that the majority of odorants recognised by one species may also be detectable by others, as suggested by Godfrey et al. [9], given that the majority of human OR subfamilies have matching parts in the other species, as is the case of the mouse [9,10], and other mammals [11].

According to Ngai and co-workers [12], the ORs, based on their amino acid sequences and phylogenetic distribution, can be classified into two classes, Classes I and II, based on amino acid sequence. Class I ORs have a tendency to bind hydrophilic odorants, and Class II ORs binds to hydrophobic odorants. The fish, teleost fish, including the goldfish, have only Class I OR genes. On the other hand, the frog as well as other semiaquatic animals, have both Classes I and II OR genes, and initially, it was thought that mammals only contained Class II ORs [13]. These results suggested that the Class I ORs are specialised in recognising water-soluble odorants, and Class II ORs recognise volatile odorants [6]. Currently, studies of the genome sequences have shown that there are a large number of Class I ORs in the human genome [14], and other mammals [11]. Indeed, 10% to 20% of the ORs in mammals are Class I ORs [10,11,14] which indicates that Class I ORs are important to mammalian olfaction skills.

Extraction of volatile and non-volatile compounds from plants and animals for the development of aromas and fragrances has been carried out since ancient civilizations and is widely used in product formulations of the cosmetic, pharmaceutical and food industries. Nowadays, the flavours used as additives in beverages and foods are now produced via chemical synthesis or extraction [15,16]. The chemical synthesis of fragrances should be avoided because this is not eco-friendly [17]. Indeed, these extraction processes lead to the formation of undesirable products on the one hand and the growing refusal of consumers to use chemicals added to food and other consumer goods on the other. Nowadays, major developments are observed in the methodologies for obtaining aromatic compounds such as microbial and enzymatic biotransformations, *de novo* synthesis and the use of genetic engineering tools [16,17,18]. Therefore, natural and sustainable processes are in growing demand from consumers, leading to the opportunity for bio-production alternatives so, novel techniques and ideas are emerging. For instance: (i) plant-cell, tissue and organ cultures (PCTOC), are an alternative for the production of high molecular weight flavouring molecules, as well as food and beverage additives [19]; (ii) metabolically engineered microorganisms and enzymatic biocatalysis are striking biotechnological options for flavour production [19]. C_13_-apocarotenoids, derived from the oxidative cleavage of carotenoids, are volatile compounds that contribute to the aromas of diverse flowers and fruits. They are used in the flavour industry and are chemically synthesised, but now they can be synthesised by bioprocess engineering [20], particularly by (iii) in situ product removal (ISPR), considered a necessary technology for the development of industrial-scale bioprocesses [21]. According to Sá and co-workers [22], enzyme-catalysed reactions are the most cost-effective strategy from the industrial point of view to reach final green products. 

Microbial cell factories, such as *Saccharomyces cerevisiae*, can be engineered using synthetic biological tools, to express synthetic pathways for the manufacturing of food and beverage flavours. Here, the biology is linked to mathematics and computer science. Systems biology, applying computational tools and mathematical models are used to comprehend complex biological networks and guide synthetic biology design [23]. 

As most active flavour compounds have a specific purpose and are delicate molecules, encapsulation of these molecules has been developed for their use. Encapsulation is effective to protect molecules, avoiding losses of actives and dissemination out of the target [24]. Still, to be able to use the label “natural flavours” and “natural fragrances”, the production of natural capsules from natural materials is required. Among biological structures, yeast cells offer good protection for active-fragrances. Microbial spores can also be used; they can fully protect actives that do not interact with each other or with the environment. Pollen grains are also able to disseminate actives in the environment [24].

## 2. Functional Characterisation and Metabolic Engineering of Flavour Compounds Biosynthesis in Plants

Several hundred volatile compounds have been identified as part of fresh and processed fruits, including fruit juices, as well as vegetables which cause varied sensory sensations during consumption [25] and determine consumer’s choice [26,27,28,29]. Even if some fruits present the same compounds known as arenes or aromatics, each fruit has its fragrance that depends on the aromatic compounds’ composition and concentration [30]. Volatile metabolites from fruits and vegetables are lipophilic, represent around 1% of plant secondary metabolites, and are mainly terpenoids, phenylpropanoids/benzenoids, fatty acid derivatives, and amino acid derivatives [27]. These compounds are important for attracting pollinators and seed dispersers and enhancing protection against abiotic or/and biotic stresses [31]. 

Multiple fruits and vegetables biochemical pathways are responsible for the volatile compounds’ composition [30]. Synthesis of aromatic compounds occurs during the fruit ripening and is influenced by several factors such as temperature and day/night variations [32]. Among all volatile compounds, esters are produced by fleshy fruit species during ripening and are the major aroma compounds in apples (*Malus domestica*), pears (*Pyrus communis*), and bananas (*Musa sapientum*). However, in soft fruits like strawberries (*Fragaria × ananassa*), volatile esters are present in a lower concentration [33].

Ripeness plays a fundamental role in volatile compounds’ biosynthesis; these compounds being often missing in immature fruits [34]. Other factors that can also influence the fruit volatile compounds concentration are the cultivar, cultural practices, and postharvest management [35].

Fruit volatile compounds present in intact tissues are classified as primary metabolites and can be classified as secondary metabolites if are produced as a result of tissue disruption due to fruit injury [36]. Therefore, the final fruit aroma profile depends on the sample preparation, namely if it is used as an intact fruit, slices or homogenised fruit samples. According to Valero and Serrano [37], volatile compounds determined in intact fruits are related to the consumer smelling perception and the fruit ripening signals, while volatile compounds determined after tissue disruption are more related to the flavour perception during fruit-eating. 

The biosynthesis of volatile compounds begins from primary metabolic routes, and the formed compounds can be classified as terpenes, fatty acid derivatives, polysaccharide derivatives, and amino acid derivatives [29]. According to Croteau and Karp [38], the volatile compounds in plants are mostly synthesised from the following pathways: (i) short-chain aldehydes and alcohols (like *cis*-3-hexenol and n-hexanal) that are synthesised by the action of lipases on lipids, followed by the action of the alcohol dehydrogenases [39]; (ii) eugenol, 2-phenylethanol, and guaiacol, resulting from the shikimic acid pathway [38]; (iii) nor-isoprenoids (like β-ionone and geranylacetone) that result from the degradation of terpenoids [40], while terpenoids can be produced by two independent pathways: the mevalonate pathway in the cytoplasm and the 2-*C*-methyl-*D*-erythritol-4-phosphate (MEP) pathway in the plastid [41]. 

How to improve the aroma without affecting other fruit or vegetable attributes is the subject of many studies. Several strategies have been developed to improve fruit and vegetable-aroma volatile compounds, either eliminating or increasing their emission. Those strategies include a wide range of targets including the metabolic pathways, hormones and transcription factors, and mechanisms involved in the storage or sequestration of volatile precursors [28,42]. The production of volatile compounds can also be influenced by lower pectins degradation in transgenic fruits by down-regulation of polygalacturonase (PG), pectin methylesterase (PME), and polygalacturonase + pectin methylesterase [28]. 

Metabolic engineering of Aromatic Amino Acids (AAA)-derived pathways, is an interesting research topic and has been used to improve the aroma and flavour of fruits, either by overexpressing an existing enzyme or by blocking a competing pathway. Manipulation of transcriptional regulation can also be used to change the metabolic profile of AAA-derived volatiles [43]. Finally, engineering of terpenoids in plants is also a hot topic that includes enhanced disease resistance, the use of allelopathic compounds in weed control and increased value of ornamentals by the production of medicinal compounds [44,45]. The fundamental factors in terpenoid engineering experiments are the phytotoxicity of the terpenoids introduced, the subcellular localization of both the precursor pool and the introduced enzymes, the activity of enzymes which modify the introduced terpenoids and the impacts on other pathways sharing the same precursor pool [44,45].

## 3. Functional Characterisation and Metabolic Engineering of Flavour Compounds Biosynthesis in Microorganism Cells

Most flavour compounds used in beverages, food or cosmetic are frequently produced by chemical synthesis causing a decrease in consumer’s acceptance due to the additions of a synthesised chemical compound to the product. Due to health awareness, the flavour companies focused their study on the biological flavour compounds, the so-called “natural flavours” or “bio-flavours” [46,47]. Plants extracts are a natural source of flavour compounds but their isolation and purification are difficult since the plant materials contain a very low concentration of the desired compounds, which makes the extraction of natural flavour compounds expensive, time-consuming and environmentally unfriendly as it requires relatively large quantities of solvents [48]. Production of natural flavour compounds from plant extract also carries severe problems for the environmental, ecological and extent of agricultural soil use. With the growing awareness of environmental sustainability and deficiency of soil, microorganisms are engineered for the biosynthesis of natural flavour compounds, linked to the advantages such as safety and enough source of precursors [49]. So, a novel promising alternative path for natural flavour synthesis is based on microorganism cell processes, i.e., fermentation or bioconversion of suitable natural precursor using microorganism cells or enzymes (biocatalysis) [44,47,50,51,52]. Microorganisms present several benefits when compared with plants, such as being fast growing, soil saving and controllable. The similar intracellular structure of yeast especially *Saccharomyces cerevisiae* with plant cells led to a widely used host for producing flavour compounds [53]. There are two ways to produce biotechnologically flavoured compounds, through de novo synthesis or by biotransformation. *De novo* synthesis is related to the production of substances using molecules such as sugars, amino acids among others which will be metabolised by microorganisms, normally generating a mixture of products in low concentration [54]. Biotransformations, is related to single reactions catalysed enzymatically, so the substrate is metabolised by the microorganism, have a higher potential for the production of flavour compounds at a commercial scale [54]. Flavour compounds produced by *de novo* synthesis uses the whole metabolism of the microorganism to produce a combination of flavour compounds, in opposite to the biotransformation, where a specific reaction(s) produced a major flavour compound [19].

The development of innovative metabolic engineering and “omic” technologies (genomics, transcriptomics, proteomics, and metabolomics) have started the way for protein and biomolecular pathway engineering. These new methodologies gave the start for very significant progress in metabolic engineering of microorganism cell factories namely for increased, terpenoids synthesis, which may be used to produce flavour compounds at an industrial scale [55]. Terpenoids (also called terpenes or isoprenoids) are obtained via the mevalonate biosynthesis pathway or the 2-*C*-methyl-*D*-erythritol-4-phosphate pathway with the former being found in yeast and constitute the two main targets of cell engineering approaches to improve terpenoid production [56,57]. To produce monoterpenes, which are produced directly by terpenoid synthase, *Escherichia coli* is extensively used for enzyme identification and for their significant activity and easy expression of these terpenoid synthases in *E. coli*, as it has a simplicity genetic manipulation [58,59,60,61,62]. Yeasts are also becoming attractive to be used, for example in engineered yeast expressing of geraniol synthase [63,64,65,66,67,68]. However, the use of the yeast cell as an efficient biosynthetic pathway has presented some limitations, such as the biosynthetic pathways are not completely explained associated to the reduced or even missing the activity of plant enzymes when expressed in yeast. In addition, reduced cell growth and reduced final products quantity could be related to declines in the native metabolic flux affected by heterogeneous pathways [69,70]. Also, the cytotoxicity of some natural product could be a limitation to using microorganism cells for producing natural flavour compounds. The identification of new microorganism hosts that can reach greater production and the development of existing strategies to identify regulatory effects in the central metabolic pathways are fundamental questions about the microorganism biosynthesis [46]. Several strategies and bio-tools to accelerate the microorganism natural products’ biosynthesis in yeast cells have been developed based on omics, metabolic engineering and protein engineering [71,72,73].

Therefore, strategies to redesign natural biosynthetic pathways are being studied by analysing the genome and transcriptome data to prediction the genes involved in the targeted compound biosynthesis, by comparing the transcriptome data between plants with high- and low-production of the target flavour compounds, numerous key genes could be predicted [72]. The low final concentration of the synthesised flavour compounds is frequently related to the reduced enzyme activity on the unnatural substrate, so strategies to improve plant enzyme activity and to enhance metabolic flux must be studied [74,75,76]. As the plant natural products biosynthetic pathways contain many steps, when hosted in the yeast cells the heterogeneous pathways would interact with the native metabolics, by competing substrates and co-factors. Therefore, this trouble will limit the targeted flavour compound production, by balancing metabolic flux distribution between heterologous pathways and native metabolic shows essential in improving the production of targeted flavour compound. Also, strategies to decrease toxicity to the hosts are needed [53,77]. 

Natural products frequently show cytotoxicity to the microorganism hosts, with the consequence of reducing cell growth and prejudice the flavour compounds production. Therefore, several strategies were studied including two-stage fermentation, pathway separation and transporters mediated compound secretion [53,77]. To lessen the negative effects on cell growth separated into two stages, in the first fermentation stage, heterogeneous pathway retains calm and cells grow fast with precursor accumulated, in the second stage, target pathway would be induced to produce the target flavour compounds. Consequently, subcellular separation is a new strategy to decrease products cytotoxicity to the microorganism hosts. An alternative strategy to decrease the internal cytotoxicity of natural products is to secrete these compounds external to the cell automatically. To attain this objective, transporters have been taken into account, for transporting the products to the extracellular space. Transporter engineering has been developed to improve the transports, due to the scarcity of transporters that can transport the flavour natural products [53,77].

**Figure 1:**
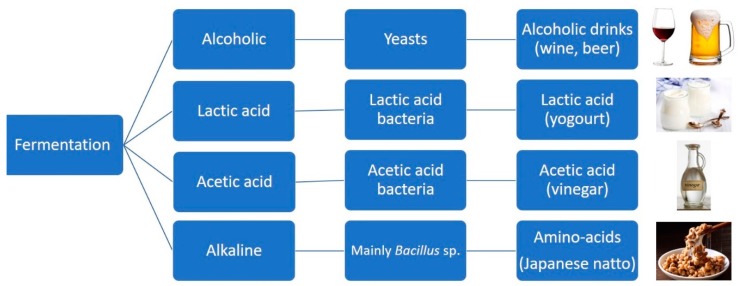
Schematic representation of the common types of fermentation, the microorganisms involved, and the end food products [79]. Alkaline-fermented foods constitute a group of less-known food products that are widely consumed in Southeast Asia and African countries. In alkaline-fermented foods, the protein of the raw materials is broken down into amino acids and peptides; ammonia is released during the fermentation, raising the pH of the final products and giving the food a strong ammoniacal smell. They can be made from different raw ingredients. For instance, Japanese natto is inoculated with a pure culture of *Bacillus subtilis* var natto [80].

Microorganisms can synthesise flavours as secondary metabolites throughout fermentation (Figure 1) using nutrients such as amino acids and sugars. This ability may be employed as an entire part of food or beverage production processes (some examples include fermented beverages like beer and wine, and fermented foods such as vinegar, yoghurt, and cheese) which defines the sensory attributes of the resulting product. It can even be used to obtain flavoured compounds that allow the product to be labelled as a “natural product”. The use of solid-state fermentation can provide higher incomes or more suitable product characteristics than submerged fermentation with inferior economic values [78,79].

Kutyna and Borneman [81] suggested that the yeast *Saccharomyces cerevisiae* acts as a microbial cell factory for the manufacturing of flavour and aroma compounds. The heterologous production of natural aroma and flavour compounds such as nootkatone, valencene, vanillin and cinnamaldehyde, is only possible due to synthetic biology. Of the thousands of flavour and aroma compounds that have been identified, however, solely a short amount has been successfully performed by heterologous means [81]. *S. cerevisiae* and *Escherichia coli* are engaged for the microbial synthesis of almost all-natural products of interest, although new microorganisms are emerging [82].

Lactic acid bacteria (LAB) are applied globally in the industrial production of fermented foods and can produce high-value metabolites associated with flavour, such as diacetyl and acetaldehyde [83]. Citrate-utilising LAB produce diacetyl during the production of butter, buttermilk and numerous cheeses, diacetyl is the typical butter aroma and extensively used in the imitation of butter and other dairy flavours when desirable in food or beverages [84]. From citrate in co-fermentation with lactose LAB produced naturally diacetyl. Escamilla-Hurtado and co-workers [85] also described the production of butyric acid, lactic acid and diacetyl in mixed cultures of lactic acid bacteria. Acetaldehyde, is another important aroma compound in dairy products, produced by LAB through diverse pathways. A main metabolic precursor for acetaldehyde synthesis by LAB is glucose through the pyruvate and acetyl coenzyme A (acetyl-CoA) intermediates of glycolysis [86]. Amino acids and other metabolites that are transformed to pyruvate can also contribute to acetaldehyde biosynthesis. The main pathway is through the conversion of threonine into acetaldehyde and glycine, a reaction catalysed by threonine aldolase [87,88]. LAB possess a small genome size (~2–3 Mb) and simple carbon metabolism [89]. These physiological and biochemistry features make these bacteria an important candidate for metabolic engineering strategies, essentially focused on the redirecting of pyruvate metabolism to produce important fermentation end-products flavour/aroma compounds such as the production of diacetyl from lactose rather than citrate has been the purpose of numerous metabolic engineering strategies [90,91,92,93,94].

## 4. Mechanisms of Olfaction and Ligand–Receptor Interaction

**Figure 2:**
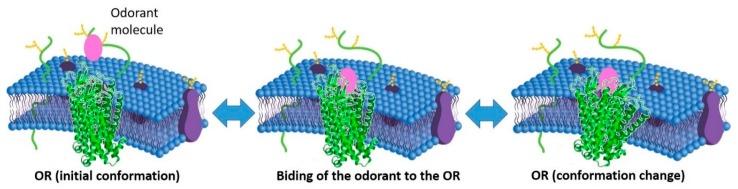
Schematic representation of the interaction between OM (odorant molecule) and the olfactory receptors. This interaction can be analysed as a sequence of 2 reversible reactions: 1st the binding of the odorant to the olfactory receptor (OR) and its consequent release; and then, 2nd, the activation (and deactivation) of the OR due to a change of its conformation.

The peripheral olfactory system was described in 1891 by Santiago Cajal and continues to elude our understanding. In this context, the publication entitled “The molecular logic of smell”, from Richard Axel, in 1995 [95] was significant. It summarises intensively the research that was done in the late 80s and early 90s, which aimed to study the molecular processes of olfactory translation that occurs in the nose´s olfactory epithelium. The olfaction consists of capturing a great amount of diverse molecular aromas of the natural world, extracting information through the personal perceptions that are related to beverages, flowers, perfumes, and whatever humans encounter, within a daily basis [96]. According to the same authors, olfaction “(…) is a chemosensory processing system that can detect potentially infinite numbers of low molecular-mass compounds, called odorants, which combine at different concentrations, to elicit this complex perception (…)” [96]. Thus, the olfactory perception results of the reversible interaction of ORs with the odorant molecules (OM) (Figure 2).

More than one “trillion” (10^12^) odours can be discriminated [97], and some authors say that “The next generation of rich media services will be immersive and multisensory, with olfaction playing a key role (…) for enhancing user quality of experience” [98]. 

In mammals, notably in humans, distinct groups of sensory neurons, which integrate several processing routes, constitute the olfactory system. This system comprises both the main olfactory system and the vomeronasal olfactory system. Each neuron in these systems expresses a type of G-protein-coupled receptor (GPCR) superfamily, specialised in the detection of a specific type of odour. Thus, the environment presents diverse odours that can be distinguished by the pattern of all sensory neurons that constitute the olfactory system [99]. For instance, humans require seven-transmembrane G-protein-coupled receptors to identify natural odorants like pheromones [100].

**Figure 3:**
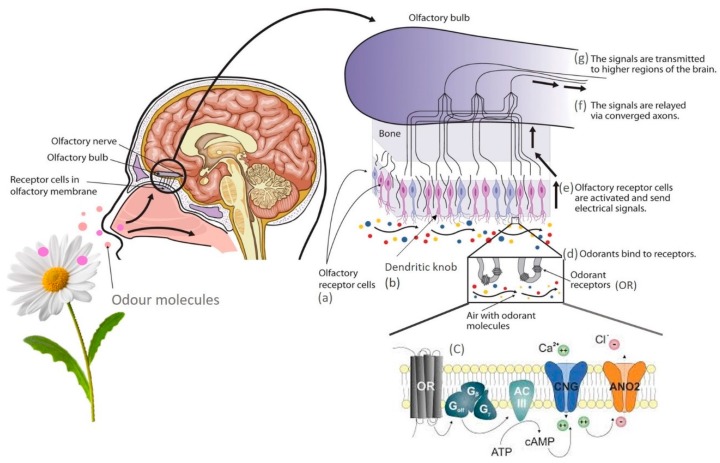
Schematic representation of olfactory transduction mechanism. OR—Odorant receptor protein; Golf—G-protein complex, made up of Gαolf (alpha (α)), Gβ (beta (β)), and Gγ (gamma (γ)) subunits; ACIII—Olfactory adenylate cyclase; CNG—Ca^2+^ Cyclic Nucleotide Gated channel; ANO2—Anoctamin Ca^2+^ activated Cl^−^ channel; ATP—Adenosine triphosphate; cAMP—Cyclic adenosine monophosphate.

But how does the transduction mechanism work? According to Villar and co-workers [101], the main olfactory epithelium or olfactory bulb comprises three main cell types, olfactory receptor cells, (Figure 3a), sustentacular cells, and basal cells. ORCs (olfactory receptor cells) project a single dendrite to the epithelial surface, where it swells forming the dendritic knob (Figure 3b).

Therefore, the initial odour detection process begins in the posterior region of the nose, when volatile molecules enter the nasal cavity (Figure 3) biding, directly or through odorant-binding proteins, to receptors on the external surface of cilia and activate receptors on the olfactory epithelium. After this binding, a complex sequence of biochemical reactions occurs, similar to those found in rod photoreceptors in the human eye, i.e., olfactory receptor neurons contain a G-protein (Golf) protein, in which its Gαolf subunit dissociates from the Gβγ complex (G beta-gamma complex) and activates a specific olfactory adenylate cyclase (ACIII) (Figure 3c), generating cyclic adenosine monophosphate (cAMP). Then is observed the neuron depolarisation because the increase of the cAMP promotes the opening of the channels and allowing cations entry, sodium (Na^+^) and mainly calcium (Ca^2+^) ions. The ensuing increase in intracellular Ca^2+^ opens Ca^2+^ activated Cl^−^ channels causing an extra inner current due to a Cl^−^ efflux amplifying the olfactory receptor potential depolarisation. This depolarisation arises from the cilia until the axon hillock region of the olfactory receptor neuron, where action potentials are generated and transmitted to the olfactory bulb [102]. The axon hillock region of the olfactory receptor neuron, the last receptor of the depolarisation that arises from the cilia, generates and transmits action potentials to the olfactory bulb [103].

Consequently, olfactory transduction can be divided into ligand binding (Figure 3d), signal generation and signal termination (Figure 3e), where the generation of action potentials conducted along the axon to the olfactory bulb. The signals are relayed via converged neurons (Figure 3f), and then transmitted to higher brain regions (Figure 3g) [102]. What happens when it is present a mixture of 2 or more odorants? Here the answer is much more complex. Several authors have done studies in this area but still, many questions remain unexplained. According to Bushdid and co-workers [97], since neither the dimensions nor the physical limits of the olfactory stimulus are known, the strategies used for other sensory modalities, namely those used regarding the estimation of the visual and auditory systems’ average resolution, are not possible to enforce in the human olfactory system. In the presence of a mixture of two odorants it is possible to determine the number of receptors activated by any concentration of the mixture, when in advance we know the affinity and efficiency of each of the components of the mixture alone, because the OR stimulated with 2 different odorants will respond with sigmoid curves as concentration’s function of the two odorants. Also, the number of ORs activated by the blend can be characterised by a logistic curve when two odorants compete for the same binding site [104,105].

Münch et al. [106] studied how mixtures of odorants interact with ORs supported by olfactory receptor neurons (ORNs) using *Drosophila melanogaster* ORNs. Their results are in agreement with others made in the rat, confirming that the response of an ORN to a binary mixture can sometimes be predicted quantitatively by knowing the ORN responses to its components. Rospars in 2013 [107] aiming to answer this question presented diverse hypotheses of the functioning of the olfactory system based on mathematical models and elementary chemical kinetics. According to the author, on one hand, whenever the concentration of the odorant molecule increases, the activated ORs follow a hyperbolic curve. On the other hand, the affinity between the odorant and the OR does not depend on the total number of ORs but the 4 reversible reactions’ rate (binding vs. release and activation vs. deactivation). Finally, this author also concluded that the maximum number of receptors activated in a given period will depend on two things: the affinity of the OR and the rate constants of the activation-deactivation reaction (but not the release of the binding reaction) [107].

## 5. Recent Sensory Analysis Techniques

### 5.1. Overview of Sensory Techniques: ‘Product Understanding’ and ‘Consumer Understanding’

Sensory evaluation of beverages is concerned with the human response to a beverage physical stimulus. For example, when one individual ingests an acid/sweet drink, the sugar molecules bind with the gustatory cells in the taste buds and the released H^+^ ion enters the tasting cells, which generates cell depolarisation and an influx of information is sent to the brain [108]. After, the brain interprets the sensations into perceptions, the stimulus is recognised and the brain expresses a response. The response can be objective “the drink is acid and sweet” or subjective, acceptance or rejection to the stimuli “I like this drink/I don’t like this drink”. The subject can also give an emotional response like “it gives me joy” or “it brings back happy memories of my summer vacations!” Sensory evaluation science focuses both on the objective measurement “product understanding” and on the subjective responses of individuals [109,110].

The sensory techniques that measure the product understanding, are well-thought-out as being objective measurements, discriminative or descriptive. Discrimination tests answer the question “Are the products similar or different?” Examples of this kind of tests are the triangle tests, duo-trio-tests or forced-choice tests [110]. Descriptive tests, qualitative and quantitative, are objective techniques once they require highly trained panellists and the degree of sensory restriction to which the sensory professional is subjected allows the reproducibility of the results that are “precise and consistent” [111].

Many of the papers published in the field of descriptive sensory tests are concerned with the development of “sensory lexicons”. The construction of these terminologies (a collection of words, called a “lexicon”), is important both for standardisation of the communication among sensory professionals working in different laboratories, and the communication of product sensory attributes to the consumers. Sensory lexicons provide a source list to describe products, including beverages (wine, beer, spirits, coffee…). Over the years, descriptive lexicons have been developed for wine [112], beer [113] and spirits [114], among others alcoholic beverages like pink port wines [115]. While the “lexicons” provide qualitative product discrimination based on attributes, the quantitative aspect is the intensity of each attribute. Different descriptive sensory methods differ in their scale usage. For example, in the QDA (quantitative descriptive analysis) method a five-point scale can be used [115], while, in other methods larger scales are utilised, such as the Spectrum™ method that use a 15 point scale with ratio properties, where 0 is no detectable amount of attribute and 15 is a high amount [113].

Sensory techniques can also be used to measure the subjective personal reaction of consumers, acceptance, liking, or consumer´s preference [109]. These methods are called “hedonic”. Also, the consumer´s perception of product benefits can be measured using semantic emotional words like “cosiness”, or “healthy” [116]. This “consumer lexicon” is important to brand identity and also for accessing product quality. However, while testing with a trained panel of assessors requires a small group of people (12–20 persons), testing with consumers, due to the person-to person variability in preferences, for achieving quantitative results that can be statistically analysed, requires a high number of participants (between 75 and 150) [109,110].

Hedonic sensory methods that measure preference and choices may be affected by food habits, attitude, and beliefs [116], culture and tradition [117], food environment and dietary [118], and, food beliefs affect the potential acceptability [118]. Nevertheless, many consumer studies have been conducted on the acceptability of commercial food products, including beverages, various consumer methods, perceptions, emotions, and cross-cultural studies [119,120,121,122,123].

#### 5.1.1. Traditional and Novel Single-Point Techniques

Reformulating a drink, like a fruit-juice, to reduce, e.g., its sugar content, will affect its consumer acceptance? Are we able to explain which sensory attributes elucidate consumer liking or healthiness product perception? Is there an easy way to approach this information? Conventional sensory analysis methods, like QDA, CATA (check-all-that-apply), flash profile, FCP (free-choice profiling), polarised sensory positioning (PSP) and holistic sensory methods (sorting, napping), have long been used to provide this information. What these methods have in common is the use of a trained or untrained sensory panel, descriptive terminology and single-point static-rating measures.

The QDA approach has been recognised and studied since the work of Stone and Sidel in 1974 [124], as an instrument for measurement and optimisation of sensory attributes of diverse food and drink products. This technique involves: (i) training the sensory panellists to quantify the specific sensory attributes of the product; (ii) produce quantitative product descriptors which can be analysed statistically [125], and represented graphically (spider graph) for perceptual mapping.

FCP belongs to the group of the descriptive analysis of sensory techniques. Each evaluator can use its descriptors and there is no need for a common vocabulary. The individual data are summarised in a plot showing the products and the attributes of each individual. Therefore, FCP does not require training making it a technique less expensive and time-consuming when compared to QDA that uses experts or trained assessors. However, since each panellist is using distinct words, the data obtained must be analysed using a generalised procrustes analysis (GPA) [126].

In the CATA methodology, assessors are asked to mark sentences/statements, previously selected. They can mark as many options as are required to express their opinion [127]. CATA is a flexible, lengthy and descriptive methodology that can be applied to the consumers with no previous training.

PSP is a technique based on the evaluation of global differences between samples and a fixed group of references or poles (for instance, similar drinks). It is one of the fasts sensory methodologies and, when compared with other methods, described above, it allows aggregation of data collected in different sensory sessions [128]. This issue is relevant when there is a need to analyse large group sets or samples with an intense sensory characteristic that can be difficult for assessors to evaluate in only one session.

As mentioned before, traditional sensory descriptive analysis is being substituted for faster sensory profiling techniques. Fleming and co-workers [129] compared the results of three fast sensory profiling techniques—CATA, sorting and PSP—using a scale of astringent stimuli. The data showed that similar plots, regardless of the method used, were obtained. 

Sorting, also called free sorting task (FST) or free multiple sorting, was first used by Lawless, and Glatter in 1990 [130] to explore the perceptual structure of odours. For performing a free sorting task only, a single session is needed. Products are randomly displayed and presented at the same time on a table with a different order per person. Tasters are asked first to sensory evaluate all the products and then to sort them into mutually-exclusive groups grounded on product-perceived resemblances. After, assessors are asked to deliver a few sensory descriptors to characterise each group that they have previously formed [131].

In 1994, Risvik and co-workers [132] published a work named “Projective Mapping: a tool for sensory analysis and consumer research”: the aim was requesting assessors to position products on a sheet of paper based on their sensory similarities. Also called Napping^®^, this fast-sensory technique uses a sheet of paper or table-top, were assessors can position the products according to their sensory similarities, producing a sensory map. Samples located closer are related and those located distant isolated are distinctive. 

However, can we be sure of the coherence of the data obtained? Dehlholm and co-workers [133], compared three fast descriptive sensory evaluation methods: free multiple sorting, Napping^®^, and flash profiling. Evaluations were carried out by diverse expert assessors from two different investigation environments and within the same time. The authors concluded that semantic results from an assessor, with no training, are dependent on the assessors’ semantic skills.

#### 5.1.2. Time-Intensity Methods

Wine tasters debate how a wine “opens in the glass after swirling”, spotting that the flavour, after the opening of the bottle, changed as a function of time. A sigh of high-quality wine is one with a pleasant and “long finish”. So, the “time profile” of a beverage is important for its sensory appeal and flavour perception.

**Figure 4:**
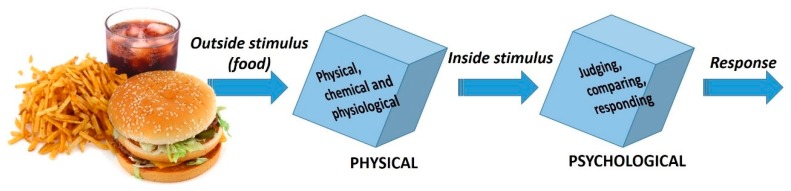
Schematic representation of the two-step model proposed to illustrate the stages in the process between flavour release/flavour perceptions. The outside stimulus is the food as put in the mouth starting physical, chemical and physiological processes. The outside stimulus is transformed into the inside stimulus by these processes. When the flavour molecules reach the receptors (OR), the neural responses are the beginning of the psychological processes. These processes include the evaluation of the strength of the stimulus, comparing it with standards, and finally, making a response. Adapted from Dijksterhuis and Piggott [134].

Flavour liberation from a food or a drink is not a unique occurrence, but an evolution of sensations and perceptions that is not constant in time. Dijksterhuis and Piggott [134] proposed, in 2000, a two-step model intended to illustrate the different stages in the process providing a framework for their integration (Figure 4). Yet, according to these authors, time plays an important role, from the moment that the drinker contacts with the drink, to the moment, after swallowing, that the sensations disappear: (1) breathing allows the transfer of the volatile compounds to the olfactory epithelium; (2) chewing also releases stimulus molecules; (3) due to loss of lubrication of the mouth surfaces, in some beverages, like wine, increases the sensation of astringency; (4) saliva movement, diluting and dissolving the drink, changes pH, wish also may permit the release of, if not, non-volatile compounds; (5) swallowing, produces a pulse of air that may contribute to the transfer of volatiles to the nasal cavity; (6) temperature variations in the mouth affecting volatility of flavour compounds [134].

Therefore, the best way of food sensory analyse is to have into account the duration of the perception stimulus. Several methods, that take into account “time” are being applied and studied. One of them is time-intensity (T-I), a sensory method that measures the intensity of a sole sensory perception, over time, in response to a single contact to a food or a drink. So, the method implies a dynamic process [135]. The objective of T-I measurements is to establish the pattern of the progress of a specific sensory characteristic. This method is ideal to distinguish products that have very diverse temporal characteristics and when measurements at a single time point are not enough. Examples may include short-term sweetness in a drink [136], or long-term astringency in wine.

Several methods for performing sensory attributes studies in time-intensity evaluations have been studied. The dual-attribute time-intensity (DATI) method [137], the temporal dominance of sensations (TDS) method [138] and, more recently, the multiple-attribute time-intensity (MATI) method [138,139].

When attributes are presented in pairs, at a given time, the DATI method can be performed. Lekalake and co-workers [140] studied an expert panel for assessing bitterness and astringency of infusions of tannin and tannin-free sorghums. In the two infusions, bitterness developed and reached extreme intensity earlier than astringency (27.9 s). The time of the astringent sensation (69.9 s) took longer than bitterness (66.3 s). Also, the temporal parameters for assessing bitterness distinguished tannin and tannin-free sorghums infusions somewhat more evidently than those for astringency. The MATI method has a similar methodology, but, with multiple stimuli. The pioneering work of Kuesten and co-workers are good examples of studies where this method was applied: Kuesten et al. [138]—“Exploring taffy product consumption experiences using a multi-attribute time-intensity (MATI) method”; Kuesten and Bi [139]—“Temporal Drivers of Liking Based on Functional Data Analysis and Non-Additive Models for Multi-Attribute Time-Intensity Data of Fruit Chews”. 

Temporal dominance of sensations (TDS) aims at gathering the sequence of the dominant sensations perceived by the panellists throughout the tasting of a product/beverage. For example, while tasting a red wine from intake to swallowing, the subject can successively perceive the attributes’ sweet and sour taste, body and viscosity, finishing with bitter taste and astringency as the dominant sensations. This method (TDS) was first proposed and studied by Pineau and co-workers, [137] in 2003. Since then, the authors have published several works, where the method is explained and compared with others [141,142].

### 5.2. Electronic Nose and Other Sensors

Electronic noses (e-noses) are devices created to recognise volatile odorous compounds. They are created to mimic the Human nose, not in his shape or size, but his ability for entrapment of odours and sensory transduction mechanisms. E-noses usually possess cross-reactive sensing arrays that upon odour exposure generate patterned responses and analytical algorithms that catalogue this patterned response [143,144].

While insects and even sharks depend on their movement through the habitat in which they live to come into contact with sensing elements, mammals can sniff, allowing contact of the odorants with the receptors, usually inside of their nasal cavities. For aroma detection by the e-nose, volatile molecules must come into physical contact with the detectors. This phenomenon occurs by contact with the detectors; or by moving the carrier air to the detector or by moving the detector through the air allowing it to contact the volatiles [143].

**Figure 5:**
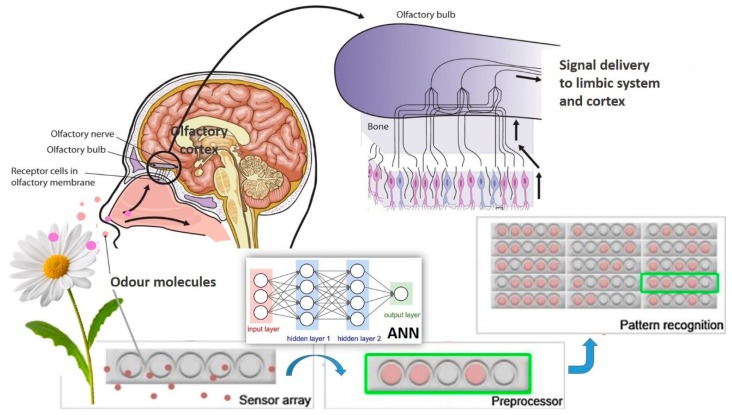
Schematic representation of an *e-nose* device, with the analogy to human nose. The electronic nose is an array of chemical sensors, connected to a pattern-recognition system that responds to odours passing over it. Different odours cause different responses that provide a signal pattern characteristic of a particular aroma. The computer evaluates the signal pattern and can compare the aromas of different samples, using pattern recognition. Artificial neural network (ANN).

In biological systems, sensing surfaces are intricate structures that consist of augmented surfaces composed by cilia or microvilli. E-nose sensing surfaces are virtually flat and uniform [143]. Yet, e-nose devices can contain up to 40 sensors, each one standardised for a precise chemical compound. Compounds and sensors combined to provide a measurement pattern. The electronic nose can only identify patterns of expected and known volatile compounds [145]. So, to be able to detect, analyse and process the information, an e-nose device must be built putting together three components, each with a specific function [146]: A sample delivery system consisting in a multisensory array; a detection system such as an artificial neural network (ANN); and a computing system with appropriated software (digital pattern-recognition algorithms and reference-library databases) [147] (Figure 5).

E-nose devices can be classified according the origin of their signal-transduction mechanisms. Several signal-transduction mechanisms have been reported: Metal oxide semiconductor (MOS) [148,149]; conducting polymer films [150]; acoustic wave devices [151]; optical transducers [152,153]; electrochemical systems [154,155] polymer film chemo-resistors [156] and quartz microbalance (QMB) sensors [157]. Regardless of the transduction mechanism, the higher the number of sensory elements in a cross-reactive sensor array (CRSA), the better is the data collected, and the specificity of the analyte identification and classification. Recently, Fitzgerald and co-workers [158] developed a cross-reactive sensor arrays to be used in e-nose devices. They developed a barcoded polymer sensor array based on porous polymer beads. Spectroscopic changes experienced by the porous spectroscopically encoded beads, after exposure to an analyte, could be used to identify and classify the marked analytes. 

Drift correction is an important concern in *e-nose* techniques and devices, for maintaining a steady performance during non-stop work. The deviations normally caused by environmental and physicochemical influences disrupt the compatibility between the gas-sensor responses and the artificial intelligence algorithms [159]. Drift component can be decomposed based on statistical characteristics by means of multivariate statistical analysis such as principal component analysis (PCA) and PCA-based component corrections [160,161] independent component analysis [162], wavelet [148] and, more recently, by active learning on dynamic clustering [159] 

Besides e-noses, science has arranged the manner of also mimic the Human tongue. The first e-tongue appeared in the 1990s. They were developed for application in ions and heavy metals analysis [163] as well as an evaluation of taste [164]. According to the International Union of Pure and Applied Chemistry (IUPAC) “An electronic tongue is a multisensory system, which consists of a number of low-selective sensors and cross-sensitivity to different species in solution, and an appropriate method of pattern recognition and/or multivariate calibration for data processing” [165,166]. An e-tongue is useful when human panels cannot/should not be used, due to beverage process circumstances; poisonous/extreme condition samples, and cost-effective reasons [167].

E-tongues can use arrangements of sensors constructed on a variety of transduction principles from electrochemical to optical or mass [168]. However, electrochemical sensors such as amperometric [169], voltammetric [170], potentiometric [171] sensors, and specially, impedimetric sensors [172] are the most usually used in beverages like wine [173], or even semi-solid food such as honey [172].

The fingerprint of a given substance can be achieved by fusion of data from different sensor types thus, allowing a better classification and identification of the analyte. This is possible due to multitransduction, i.e., by measuring different properties of the same sensor. One example is the quantification of electrochemical and optical changes observed for porphyrin electro-polymer films [174]. It is also possible to merge data from physically separated optical and electrochemical sensors as has been studied by Gutiérrez and co-workers [175] for quality control of wine. In recent years, the inclusion of biosensors with specificity to certain compounds in the array has been investigated. These coupled devices are called bioelectronic tongues (bio-tongue) and are able to provide global information about the beverage or food products, whereas, simultaneously, provide data about a particular compound, due to the specificity of the biosensor [168,176].

With the purpose of replacing human tasters, e-tongue devices can be combined with sensors for gaseous samples, e-noses [177,178], and even with computer vision for colour and surface characteristics evaluation [179]. In 2011, Cole and co-workers [180] created a joint electronic tongue and nose for a flavour-sensing system. Flavour is understood as being the joint experience of oral and orthonasal stimulus, a combination of the human gustation and olfaction. So, the system proposed contained both an e-tongue made with a shear horizontal surface acoustic wave (SH-SAW) sensors, and an e-nose based on chemFET sensors (chemically-sensitive field-effect transistor sensors). They analyse the liquid phase and the gaseous phases, respectively [180]. But, can we call this sensor an e-flavour?

## 6. Recent Innovations in the Statistical Technique of Sensory Data Analysis

Statistical techniques play an important role in sensory data analysis. The need to determine trends, relationships among variables, similarity among products or consumers, are examples of the type of problem that statistical techniques may need to be applied. During a long period, basic statistical techniques such as statistical hypothesis testing were all that would be required in sensory data analysis. Over the last few decades, the new requirements facing industry are gradually changing the sensory role into a more active one, responsible for creating new product ideas based only on sensory characteristics. The use of different kinds of measuring devices, more rigorous scales to measure perceptions, new sensory tests and increased interest in all the sensory properties of consumer products led to the need for more sophisticated statistical techniques capable of handling multiple variables simultaneously and being able to withdraw the information they provide. Moreover, advances in computational technologies allow the development of statistical packages capable of analysing sets of multivariate data. As a consequence, multivariate statistical techniques are now increasingly being used in sensory data analysis.

There are several multivariate techniques, and the choice of what is appropriate depends on the type of data and the objectives of the study. All of these techniques require statistical assumptions. So, we must always be sure that the method used is the most suitable in the specific case, otherwise, the results obtained may not be reliable. Among the most common techniques used for identifying sensory characteristics in wine and food are: PCA [181,182]; multivariate analysis of variance (MANOVA) [183], cluster analysis [182], correspondence analysis (CA) [184]; multidimensional scaling (MDS), [182], hierarchical cluster analysis (HCA) [185], partial least squares (PLS) regression [186,187], multiple linear regression (MLR) [187], GPA, a method used in sensory data analysis previously, and preference mapping to decrease the scale effects and to achieve a consensus configuration, allowing to relate the closeness between the terms that are used by diverse experts to explain products [188]; and, more recently, non-parametric MANOVA [189] with categorical PCA (CATPCA) [115,190]. CATPCA is appropriate to reduce the dimensionality of qualitative variables (measured on a nominal or ordinal scale) to a lesser number of uncorrelated variables (principal components) that characterise most of the information of the original variables. This technique, generally referred to by optimal scaling assigns numerical quantifications to the categories of each of the qualitative variables. The main advantages of CATPCA are its ability to deal with non-linear relationships and jointly analyse numeric, ordinal, and nominal variables [187]. 

In 2018 Vilela and co-workers [191] applied an innovative multivariate analysis technique, structural equation modelling (SEM) for identifying wine sensory characteristics of monovarietal wine from the Portuguese Vinho Verde wine Demarcated Region. SEM is a statistical technique used to decrease the number of perceived variables (sensory descriptors) into a lesser number of latent (not observed) variables by examining the covariances between the observed variables [192,193]. This data analysis technique that can be viewed as a combination of factor analysis and multiple regression analysis had its origin at the beginning of the 20th century, from the important works of Charles Spearman [194], an English psychologist known to work in statistics, as a pioneer of factor analysis, and for Spearman’s rank correlation coefficient. Structural equation modelling is a general and flexible framework for data analysis that includes traditional techniques such as regression analysis, factor analysis, confirmatory factor analysis, path analysis, and discriminant analysis. 

## 7. Final Remarks

The increased consumer preference for natural and sustainable products makes the production of natural flavours, which define the sensory perception of beverages and other food products, an ever-challenging purpose for academic and industrial investigation. A consumer’s choices and demands have triggered an evolution in the world of flavours. From simple extraction methodologies to engineered metabolic routes, scientists have become experts in mimicking nature and, in some cases, compete to obtain better, natural, ambient friendly and flavorous products.

The advances are not only in terms of chemistry, biochemistry, microbiology or sensory analysis, but also mathematics, computer and electronic help the flavour development technologies that allow faster, safer, economic and precise aroma/flavours compounds synthesis, analysis, characterisation, and quantification. Nowadays, major developments are observed in the methodologies for obtaining aromatic compounds such as microbial and enzymatic biotransformation, de novo synthesis and the use of genetic engineering tools. Microbial cell factories, such as *Saccharomyces cerevisiae*, can be engineered using synthetic biological tools, to express synthetic pathways and systems biology, applying computational tools and mathematical models, used to understand complex biological networks, and guide synthetic biology design. Therefore, the choice for natural and sustainable processes is growing, leading to the opportunity for bio-production alternatives.

Sensory techniques with the use of trained or untrained panellists have also evolved from traditional multiple points and structured scales techniques to novel single-point techniques and time-intensity methods. Conventional sensory analysis methods, like QDA, CATA, flash profile, free choice profiling, polarised sensory positioning and holistic sensory methods, have long been used to provide the beverages and food products sensory information. However, several methods, that take into account “time” are being applied and studied. Time-intensity (T-I), methods or temporal dominance of sensation methods have been useful, once they are ideal to distinguish products that have very diverse temporal characteristics and when measurements at a single time point are not enough.

Sometimes, the use of a panel of tasters is not enough. Humans can be tired and some subjectivity is implied in their evaluation, or the products are not yet ready to be tasted by humans. To circumvent the limitations of the human being, science has arranged a manner of also mimicking the human nose and tongue. Electronic noses (e-noses) are devices created to recognise volatile odorous compounds. They possess cross-reactive sensing arrays that upon odour exposure generate patterned responses and analytical algorithms that catalogue this patterned response. E-tongues also use arrangements of sensors constructed on a variety of transduction principles from electrochemical to optical or mass.

However, machines, like humans, are also not one 100% reliable. Drift correction is an important concern in e-nose and e-tongue techniques and devices, for maintaining a steady performance during non-stop work. The deviations disrupt the compatibility between the sensor’s responses and artificial intelligence algorithms. The drift component can be decomposed based on statistical characteristics employing multivariate statistical analysis (PCA, independent analysis, wavelet, and active learning on dynamic clustering). Some of these statistical methods are also used to treat data obtained by traditional sensory techniques, using human panellists.

However, strangely, in terms of electronics, it would be expected that a fully developed e-flavour system would be on the market, ready to be used for the many industries (food and wine) that work with flavour perception and consumer preference with the aim of producing flavorous products. Perhaps there is a limit on what man can achieve; or perhaps it is not as easy as it seems to mimic the human perception that we call “flavour”.
